# The Effects of Diverse Interventions on Diabetes Management Among Arabs With Diabetes: A Systematic Review

**DOI:** 10.1111/jan.16423

**Published:** 2024-09-05

**Authors:** Omaima Abd Elqader, Einav Srulovici

**Affiliations:** ^1^ The Cheryl Spencer Department of Nursing University of Haifa Haifa Israel

**Keywords:** Arabs, diabetes mellitus, health behaviour, health promotion, program evaluation, self‐management

## Abstract

**Aim:**

To identify, describe, and critically evaluate the effects of various interventions on diabetes management outcomes among Arabs with diabetes.

**Design:**

A systematic review.

**Data Sources:**

The search was conducted across three databases: PubMed, CINAHL and the Cochrane Collaboration in December 2023.

**Review Methods:**

Screening involved randomised controlled trials and nonrandomised studies that focused on the effects of interventions on diabetes management among Arab with diabetes. The Preferred Reporting Items for Systematic Reviews and Meta‐Analyses (PRISMA) checklist guided the review process. Two researchers independently applied eligibility criteria. Data extraction captured key study details, and methodological quality was assessed using Downs and Black's checklist. This review is registered with the International Prospective Register of Systematic Reviews (PROSPERO; registration number CRD42024555668).

**Results:**

Thirty‐five articles were reviewed, yielding 65 outcomes. Effective interventions included personalised care, patient‐centred education and direct patient contact through lifestyle modifications, advice, feedback, motivational conversations and calls. These approaches improved haemoglobin A1c, fasting blood glucose, physical activity and medication adherence. Conversely, nonpersonalised remote monitoring and social media interventions showed no significant improvements. Notably, tailored nutritional and physical activity advice positively impacted body mass index and systolic blood pressure among Arab women with diabetes.

**Conclusion:**

The findings underscore the effectiveness of personalised care and direct patient contact in optimising diabetes management among Arabs with diabetes.

**Impact:**

This review highlights the importance of prioritising direct patient contact over remote methods such as social media in interventions on diabetes management among Arabs with diabetes. It emphasises the need for culturally sensitive approaches, particularly for women.

**Patient or Public Contribution:**

No patient or public contribution, as this study constitutes a review of existing research.

**Summary**

- Personalised diabetes management interventions, including individualised lifestyle self‐management and patient‐centred education, have significantly improved objective and subjective outcomes among Arabs with diabetes.
- Traditional face‐to‐face interventions, incorporating motivational conversations, feedback and personalised advice, are more effective than social media interventions for diabetes management among Arabs with diabetes.
- Culturally tailored interventions, particularly for Arab women with diabetes, are needed to address specific barriers and improve diabetes‐related outcomes, such as weight loss and treatment adherence.

## 1 Introduction

Diabetes mellitus constitutes a significant global health challenge, with prevalence reaching alarming levels worldwide, affecting approximately 9.8% of adults aged 20–79 years, totaling over half a billion individuals globally (Cho et al. 2021). The prevalence of diabetes varies across societies, influenced by behavioural and sociodemographic factors (Sharkia et al. 2019). Notably, Arabs exhibit elevated rates of diabetes, such as an 18% prevalence among American Arabs aged 20 to 75 years, surpassing the 8.2%–11.9% rates observed in the rest of the U.S. population (Saadi et al. 2007). Additionally, Kuwait stands out with the third‐highest global prevalence of diabetes at 24.9%, ranking behind Pakistan and French Polynesia with rates of 25.2% and 30.8%, respectively (Cho et al. 2021).

Rapid urbanisation, sedentary lifestyles and dietary shifts driven by economic growth are pivotal factors fuelling the escalating incidence of type 2 diabetes across Arab nations (Khan et al. 2021; Sharkia et al. 2019; Zhou et al. 2016). These transformations have facilitated the proliferation of Western‐style fast‐food establishments, increased mechanisation and the availability of inexpensive labour, all contributing to a burgeoning diabetes epidemic (Khan et al. 2021; Sharkia et al. 2019).

Achieving optimal metabolic glucose control is crucial for preventing microvascular and macrovascular complications among patients with diabetes (Bekele et al. 2020; Khan et al. 2021). Despite the array of pharmacological options available, including insulin, sulfonylureas and metformin, lifestyle modifications remain indispensable in diabetes management (Elkhalifa et al. 2024a). These modifications encompass comprehensive strategies such as diabetes self‐management education, medical nutrition therapy, physical activity promotion and psychosocial support, all crucial for improving health outcomes (American Diabetes Association [ADA] 2018; Powers et al. 2020).

Studies have shown that adherence to modified lifestyle, diet and pharmacological treatment is associated with improved glycaemic control, reduced emergency department visits, decreased hospitalisations, lower medical costs and improved health outcomes for patients (Al‐ma'aitah et al. 2022; Krass, Schieback, and Dhippayom 2015; McSharry et al. 2016). However, evidence suggests that adherence rates among patients with diabetes are less than ideal (Krass, Schieback, and Dhippayom 2015), with an estimated 1 in 3 patients not taking their diabetes medication as prescribed, particularly among racial/ethnic minorities and individuals with low socioeconomic status (Althubyani et al. 2024; Love, Peter, and Julie 2022; Nelson et al. 2018). Thus, understanding and addressing these barriers—ranging from individual to systemic and cultural factors—are imperative in developing effective diabetes management strategies tailored to Arab populations (Bekele et al. 2020; Kvarnström et al. 2021).

Cultural norms significantly influence diabetes management among Arabs, shaping dietary practices and perceptions of chronic disease (Levin‐zamir et al. 2015). These cultural dynamics, coupled with socioeconomic disparities, underscore the complexity of diabetes care in this demographic (Sweileh et al. 2014). Recognising these influences, interventions examining the effects on diabetes management among Arabs can be challenging and must carefully address these specific barriers to be effective (Sapkota et al. 2015). The American Diabetes Association emphasises culturally sensitive diabetes self‐management education and support to optimise outcomes among Arab Americans, highlighting the importance of tailored approaches (ADA 2018; El Masri et al. 2020).

While lifestyle interventions have shown promise in lowering haemoglobin A1c (HbA1c) levels among diabetes patients globally (García‐Molina et al. 2020), their effectiveness appears variable among Arabs (Bertran et al. 2021; Kreidieh et al. 2017). Conversely, adherence to antidiabetic medications and glycaemic control was less observed in the general population (Sapkota et al. 2015), but appears effective in the Arab population (El‐Awaisi et al. 2022; Gillani et al. 2021). Moreover, adoption of regular physical activity and personalised self‐management strategies have been associated with improved glycaemic control in Arab populations, highlighting their critical role in diabetes management (Al‐ma'aitah et al. 2022; Althubyani et al. 2024). Moreover, interventions that consider cultural and religious practices, such as addressing the unique challenges faced by Arabs during the Ramadan fasting month, have also shown encouraging outcomes (Srulovici et al. 2019). Therefore, emphasising these components is crucial to mitigate diabetes‐related complications and enhance overall quality of life in Arabs with diabetes.

The variability in intervention effectiveness underscores the need for a systematic review of studies evaluating the effects of interventions on diabetes management specifically for Arabs. Such a review will facilitate the identification of effective strategies and their contextual factors, laying a foundation for future research and guiding healthcare providers in designing targeted interventions for this population.

## 2 The Review

### 2.1 Aim

The aim of this systematic review was to identify, describe and critically evaluate the evidence presented in published articles that investigate the effects of interventions on diabetes management among Arabs with diabetes.

### 2.2 Search Methods

The systematic review utilised three electronic databases: PubMed, CINHAL and Cochrane Library. Detailed search strategies for each database are outlined in Appendix S1. The search period encompassed all available years of publication. The objective was to identify all research studies evaluating interventions aimed at improving diabetes management among Arab patients. Duplicate references from different databases were eliminated, and a manual review of reference lists from identified studies was conducted. This systematic review adhered to the Preferred Reporting Items for Systematic Reviews and Meta‐Analysis (PRISMA) 2020 checklist (Page et al. 2021), and it was conducted following a prespecified protocol registered with the International Prospective Register of Systematic Reviews (PROSPERO) (registration number CRD42024555668).

### 2.3 Inclusion and Exclusion Criteria

Inclusion criteria included (1) *Population*: Arab individuals aged 18 years or older with diabetes, prediabetes and/or gestational diabetes; (2) *Intervention*: Studies focusing on interventions that examine the effects on diabetes management; (3) *Control*: No requirement for a comparison group; studies employing pre–post designs with a single group were included; (4) *Outcome*: No restriction on outcomes; all outcomes available in the identified studies were included; and (5) *Study Design*: Randomised controlled trials (RCTs) and nonrandomised studies. Eligible studies had to be peer‐reviewed journal articles published in either English or Arabic.

Exclusion criteria involved studies collecting data at a single point in time without follow‐up, presenting observations on individual cases, analysing data at the population level rather than individual‐level data, summarising existing literature without presenting original research findings and nonresearch articles expressing personal viewpoints or opinions. These criteria ensure that the included studies provide robust and relevant pre–post outcome data on interventions for diabetes management in Arab populations.

### 2.4 Study Selection and Literature Search Process

**FIGURE 1:**
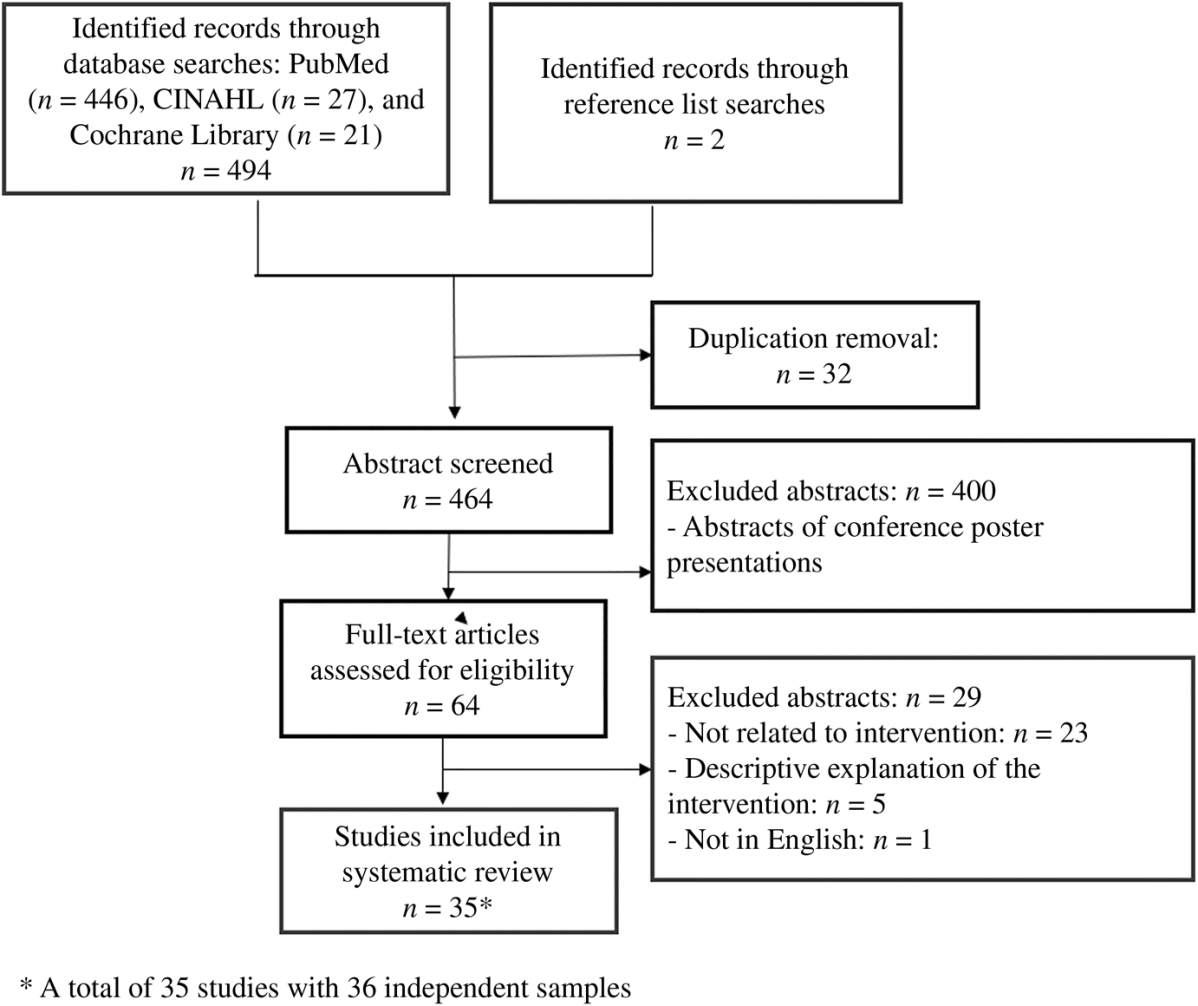
Flow chart of the study selection process.

Initially, a total of 494 papers were retrieved, from which duplicate references were removed, leaving 464 unique papers for initial screening. Titles and abstracts of these papers were screened against predefined inclusion criteria, resulting in 64 papers deemed potentially relevant for full‐text review (Figure 1). Next, full‐text articles of the 64 identified papers were reviewed in detail. Among these, 23 papers were excluded for lack of relevance to interventions aimed at fostering a healthy lifestyle and improving diabetes outcomes among Arab patients with diabetes. An additional five papers were excluded as they solely provided descriptive explanations without any evaluation component, and one paper was excluded due to being non‐English. To supplement database searches, a manual review of reference lists within the included articles was conducted, identifying two additional studies that met inclusion criteria. The final selection of studies provided a comprehensive representation of various study designs, target populations, settings, interventions, postintervention follow‐up durations, summary measures and quality assessment scores, all detailed in Table 1.

**TABLE 1:** Summary of studies examining the effects of interventions on diabetes management in Arabs with diabetes.

Author, year	Country	Target group	Sample size	Gender	Mean age (years)	Setting	Study Design	Intervention type	Intervention content	Intensity of intervention^a^	Time of follow‐up	Intervention conductor	Quality assessment
Abaza, Marschollek, and Schulze 2017	Egypt	T2D	73	All	Int. 51.24 ± 8.66 Cont. 51.77 ± 9.68	Hospital	2‐arm RCT	Educational	Diabetes care text messages	Daily messages and weekly reminders. Over 3 months	3 months	Researcher, physicians, nurses	21
Abduelkarem and Sackville 2008	UAE.	T2D	75	All	51 ± 11.3	Community pharmacies	Quasi‐experiment 1 group pre–post	Educational	Diabetes self‐care by reminders packages to every patient	12 sessions, weekly, for 3 months	3,6,24 months	Community pharmacies	16
Agbaria^b^ et al. (phase 1) 2020	Palestine	Arab women	50	Females	Int. 48.4 ± 12.7 Cont. 57 ± 11.3	Community centres	Quasi‐experiment two‐group pre–post	Educational, exercise and nutrition	Community‐based, culturally adapted, health promotion intervention	12 sessions, 3 h each, (2 h lecture and 1 h exercise), for 12 weeks	6 months	Nurses	17
Agbaria^b^ et al. (phase 2) 2020	Palestine	Arab women	117	Females	NI	Community centres	Quasi‐experiment single‐group pre–post	Educational exercise and nutrition	Community‐based, culturally adapted, health promotion intervention followed by ‘WhatsApp’	12 sessions, 3 h each (2 h lecture and 1 h exercise) and messaging mobile after intervention, for 12 weeks	6 months	Nurses	17
Al Hayek, Robert, and Al Dawish 2021	SA	T2D	105	All	45.1 ± 7.90	Diabetic centre	Quasi‐experiment single‐group pre–post	I‐Port Advance system	Use of the Free Style Libre	1 session and scans performed over 3 months	3 months	Physician, diabetes educator	19
Al Mazroui et al. 2009	UAE	T2D	240	All	Int. 48.7 ± 8.2 Cont. 49.9 ± 8.3	Hospital	2‐arm RCT	Pharmaceutical and educational	Pharmaceutical and healthy lifestyle, diabetes management, with personal contact	Monthly personal advice	4,8,12 months, 10 years	Clinical pharmacist, physicians, nurses	21
Al‐Adsani et al. 2008	Kuwait	T2D and T1D	250	NI	NI	PHC	Quasi‐experiment single‐group pre–post	Educational	Clinical guidelines, workshops and training, clear management structure, audit and feedback for team	4 audits annually	13,24,36 months	Physicians, nurses	12
Al‐Arifi and Al‐Omar 2018	SA	T2D	174	All	52.9 ± 14.2	Hospital	Quasi‐experiment single‐group pre–post	Educational	Medications, nutrition, self‐monitoring and management, telephone follow‐up service	4 sessions^a^	3,6,12 months	Multidisciplinary team	12
Al‐Bannay et al. 2015	SA	T2D or with risk of diabetes	35	Females	NI	PHC	Quasi‐experiment two‐group pre–post	Educational	Lifestyle behaviour adapted to the cultural contexts	NI sessions, for 6 weeks	6 months	NI	13
Albikawi, Petro‐Nustas, and Abuadas 2016	Jordan	T2D	149	All	51.40 ± 6.80	PHC	Quasi‐experiment two‐group pre–post	Educational	Diabetes self‐care management booklet, DVD viewing, counselling rehearsal session and a telephone follow‐up	3 sessions (2 DVD lasting 17–40 min)	2 weeks, 3 months	Physicians, nurses	19
Alfawaz et al. 2018	SA	Prediabetes	294	All	Int.(A) 43.4 ± 7.8 Int.(B) 42.6 ± 6.9 Cont.42.3 ± 11.2	Hospital	3‐arm RCT	Pharmaceutical and educational	General advice (GA) on lifestyle change; INT. A—intensive lifestyle modification individual consultation with the dietician; INT.B—GA + metformin	1 orientation session + seminars of lifestyle modifications every 4 months^a^	6,12 months	Physicians and dieticians	24
Al‐Haj Mohd et al. 2016	UAE	T2D	446	All	Int. 62 ± 11.0 Cont. 61 ± 11.0	PHC	2‐arm RCT	Educational	Medication adherence and compliance	Session of 30 min, weekly phone call for 3 months^a^	6 months	Nurses	22
Al‐Hamdan et al. 2019	SA	Prediabetes	190	Females	40.6 ± 9.8	PHC	2‐arm RCT	Educational , nutrition and PA advise	One‐on‐one intensive lifestyle modification, dietary counselling	Minimum 6 sessions, NI for time, for 3 months	3,6 months	Dietitian	21
Al‐Hamdan et al. 2021	SA	Prediabetes	253	Females	Int.(A) 42.9 ± 12.2 Int.(B) 43.7 ± 8.1 Cont. 50.9 ± 7.1	Hospital	3‐arm RCT	Education, nutrition and PA advise	Modification through social media or frontal	6 sessions, twice a month, during 3 months	3,6 months	Dietitian	22
AlHaqwi et al. 2023	SA	T2D	130	All	58 ± 8.1	Diabetic centre	Quasi‐experiment single‐group pre–post	Educational	Patient‐centred and self‐care education	NI sessions, initial 30–45 min patient‐centred educational and another 10 h during 6 months	3,6 months	Family physicians, nurses, pharmacists, diabetes educators and other support staff	19
Alibrahim et al. 2021	Kuwait	T2D	291	All	56.8 ± 10.01	PHC	Quasi‐experiment two‐group pre–post	Educational	Self‐management	4 sessions of 1 h each, further sessions depend on patients' needs^a^	6,12 months	Physicians, Certified Diabetes Educator, nurses	22
Al‐Ofi et al. 2019	SA	GDM	57	Females	Int. 32.5 ± 5.8 Cont. 32.4 ± 5.3	Hospital	2‐arm RCT	Telemonitoring device	Suppling telemonitoring device and application monitor blood sugar and weight gain	1 session and installed application with a contract for 12 months	24–28 weeks of gestation, 6 weeks postdelivery	Diabetic care team	20
Al‐Shookri et al. 2012	Oman	T2D	170	All	50.7 ± 10.4	Hospital	2‐arm RCT	Educational	Nutrition prescription, eating and exercise goals	4 sessions (1 session 1–1.5 h)2,3 sessions (30–45 min) during 1–1.5 months, 4 session every (6–12 months)	3,6 months	Dietitian	20
Ba‐Essa et al. 2015	SA	T2D and T1D	60	All	Int. 50.80 ± 15.52 Cont. 48.97 ± 14.21	Diabetes Clinic	Quasi‐experiment two‐group pre–post	Educational	Management, self‐care and monitoring, food items, exercise	4 sessions, monthly^a^	4 months	Researchers and a health education	20
Baynouna et al. 2010	UAE	T2D and/or HTN	4947	All	NI	PHC	Quasi‐experiment single‐group pre–post	Educational	Audit feedback, administration, decision making aids, system change, Information, self‐management	NI	12,24,36 months	Physicians, nurses and pharmacist	11
Haddad et al. 2014	Iraq	T2D	50	All	51.4 ± 10.3	Hospital	Quasi‐experiment single‐group pre–post	Educational	Diabetes self‐management by message services	Weekly messages, for 7 months	6 months	NI	12
Istepanian et al. 2014	Iraq	T2D	12	NI	Int. 54.8 ± 12.7 Cont. 55.2 ± 10.1	Hospital	Feasibility 2‐arm RCT	Educational	Mobile diabetes system for self‐monitoring (remote web)	NI^a^ sessions, for 3 months	6 months	Healthcare providers	13
Jarab et al. 2012	Jordan	T2D	171	All	Int. 63.4 ± 10.1 Cont. 65.3 ± 9.2	Hospital	2‐arm RCT	Educational	Pharmaceutical care, educational and lifestyle management, motivational interview	NI sessions, followed by 8 weekly telephone follow‐up calls	6 months	Clinical pharmacist, physician	22
Jenhani et al. 2005	Tunisia	T2D and T1D	87	All	53 ± 14.04	PHC	Quasi‐experiment single‐group pre–post	Educational	Diabetic care, patient problems	6 monthly sessions^a^	6 months	General practitioners and nurses	13
Khattab et al. 2007	UAE	T2D and T1D	2548	All	55.3 ± 11.6	PHC	Quasi‐experiment single‐group pre–post	Educational	Diabetic care Identified barriers. Suggested solutions group discussion sessions	NI	22 months	Multidisciplinary team	14
Mahmoud et al. 2018	SA	T2D	99	All	NI	PHC	Quasi‐experiment single‐group pre–post	Psychoeducational	Diabetic care, self‐care, lifestyle modifications and good compliance with medication individual experience and feedback	4 sessions, 3 h weekly for 4 weeks	5 months	Nurse and psychoeducation	17
Mohamed et al. 2013	Qatar	T2D	430	All	Int. 52 ± 8.9 Cont. 55 ± 10.7	PHC and Hospital	2‐arm RCT	Educational	Patient‐centred educational booklet for diabetic self‐management, individual experience and feedback	4 sessions, 3–4 h each, NI for duration	12 months	Health Educators	21
Munsour et al. 2020	Qatar	T2D	140	All	54 ± 13.0	Hospital	2‐arm RCT	Educational	Tailored consumer medicine information intervention	3 sessions, of 30–40 min each^a^	3,6 months	Physicians, pharmacists, nurses	20
Rashed et al. 2016	Palestine	T2D	215	All	51.07	Diabetes Clinic	Quasi‐experiment single‐group pre–post	Educational	Diabetic self‐care, dietary, physical activity	NI for sessions, 4 h, NI for duration	NI	Researcher	16
Reed et al. 2001	UAE	T2D and T1D	219	All	Int. 49.4 ± 11.7 Cont. 53.6 ± 10.9	PHC	Quasi‐experiment two‐group pre–post	Educational	Multifaceted, diabetic nutrition and care, motivational interview	NI sessions, for 18 months	12 months	Physicians, nurses, nutritionist	20
Reed et al. 2005	UAE	T2D and T1D	738	All	Int. 53.3 ± 10.9 Cont. 54.1 ± 10.4	PHC	Quasi‐experiment two‐group pre–post	Educational	Multiple modalities, diabetic care, adherence to guidelines.	NI sessions, during 33 months	12 months	Physicians, nurses, nutritionists	20
Sadiya, Abdi, and Abusnana 2016	UAE	T2D and/or obesity	45	Females	42 ± 9.0	Diabetes Clinic	Quasi‐experiment single‐group pre–post	Educational, nutrition and PA advice	Lifestyle intervention, individual dietary modification, PA, behavioural therapy	8 sessions, for 3 months	3,12 months	Dietitians, therapist, nurse physicians	17
Shehab, Elnour, and Abdulle 2012	UAE	T2D	254	All	49 ± 2.1	Hospital	Quasi‐experiment single‐group pre–post	Educational	Diabetes care, lifestyle modification, self‐monitoring, decision making process, medical administration	NI^a^	3,6 months	NI	18
Utz et al. 2018	Morocco	GDM	210	Females	Int. 27.9 ± 6.5 Cont. 27.3 ± 6.7	PHC	2‐arm RCT	Educational	Counselling on nutrition and exercise	NI^a^ sessions, before 24 weeks or in the second trimester	After diagnosis weekly or twice monthly, up to 8 weeks postpartum	Nurse and care providers	22
Wani et al. 2020	SA	Prediabetes	300	All	Int. 43.10 ± 9.4 Cont. 43.75 ± 10.9	PHC	2‐arm RCT	Educational	Intensive modifying lifestyle	4 sessions, 3–4 h each, for 4 months	6,12 months	Dietician and a physician	21
Wishah, Al‐Khawaldeh, and Albsoul 2015	Jordan	T2D	106	All	Int. 52.9 ± 9.6 Cont. 53.2 ± 11.2	Hospital	2‐arm RCT	Pharmaceutical care and educational	Medical administration adherence to diabetes self‐care and to medication, telephone calls	NI^a^ sessions, 30 min with pharmacist after seeing the physician	6 months	Pharmacist and physician	11

Abbreviations: GDM, gestational diabetes mellitus; h, hour; NI, no information; PA, physical activity; PHC, primary health care; RCT, randomised controlled trial; SA, Saudi Arabia; T1D, type 1 diabetes; T2D, type 2 diabetes; UAE, United Arab Emirates.

^a^ Intensity of intervention includes number of sessions, time for each session and duration of intervention.

^b^ A study with two independent samples.

Finally, a total of 35 studies were selected for inclusion, encompassing 36 independent samples (with one study including two different samples). Throughout the selection process, two independent reviewers rigorously screened titles, abstracts and full‐text articles, and independently extracted data from the included studies. Any discrepancies or disagreements were resolved through discussion, with the involvement of a third senior reviewer when consensus could not be immediately reached.

### 2.5 Quality Appraisal

The methodological quality of the included studies was assessed using Downs and Black's (1998) checklist, a widely recognised tool for evaluating RCTs and nonrandomised studies. This checklist systematically evaluates measures implemented to mitigate bias and errors in study design, execution and result analysis. It consists of 27 items covering critical domains, such as reporting, external validity, internal validity and statistical power of clinical trials. Each item was scored on a binary scale of 0 or 1, except for one item that allows a score of 0, 1 or 2 to assess the presence of principal confounders (no, partially or yes, respectively). Consequently, the maximum adjusted score achievable in this review was 28 points, ensuring a comprehensive assessment of study quality and robustness.

### 2.6 Data Extraction

A standardised data extraction form was meticulously developed to comprehensively capture information from each included study. The extraction form encompassed essential variables such as author names, year of publication, country of study, target group characteristics, sample size, gender distribution and mean age of participants. Additionally, details regarding the study setting, specific study design employed (e.g., RCT and quasi‐experimental two‐group pre–post) and the nature of the intervention were systematically documented. The form also included intricate aspects of the interventions, delineating the intervention type, content, intensity and duration of follow‐up periods. Information on who conducted the interventions and how they were delivered was meticulously recorded. In terms of outcomes, data extraction was based on the information available in the reviewed studies, encompassing a wide range of measures reported by each study without predefined specifications. This approach ensured a comprehensive overview of the interventions and their reported effects across the included literature. Finally, a detailed assessment of study quality was undertaken, evaluating methodological rigour and potential biases using established criteria.

### 2.7 Data Synthesis

The decision to opt for a narrative synthesis approach instead of conducting a meta‐analysis stemmed from the significant heterogeneity observed in the implementation of interventions, as well as the diverse durations and lengths of follow‐up across the included studies. Moreover, the wide variation in outcome measures utilised among the studies further justified the choice of narrative synthesis. Additionally, the study encompassed various research designs beyond RCTs, including quasi‐experimental two‐group pre–post and one‐group pre–post studies. These variations could potentially impact the integrity or reliability of the findings. Therefore, in this approach, pertinent outcomes from each study were meticulously identified and subjected to a comprehensive narrative synthesis. The synthesis was structured into three distinct categories: objective measures (i.e., laboratory measures, physical examination, adherence to diabetes guidelines and diabetes‐related cardiac complications), subjective measures (i.e., patient‐reported outcomes and lifestyle modification) and women‐related measures (i.e., outcomes for women with prediabetes or diabetes and gestational diabetes mellitus outcomes), enabling a thorough evaluation of the collected findings.

## 3 Results

### 3.1 Study and Intervention Description

The included studies in this systematic review were conducted between 2001 and 2023, with a majority of them being conducted in Saudi Arabia (SA) (*n* = 11) and the United Arab Emirates (UAE) (*n* = 9). Among the studies, the majority focused on patients with type 2 diabetes (*n* = 19), while others examined both type 1 and type 2 diabetes (*n* = 5), prediabetes (*n* = 4), gestational diabetes (*n* = 2) and participants with type 2 diabetes and/or obesity and hypertension (*n* = 2). The sample sizes of these studies ranged from 12 to 4947 subjects. Specifically, five studies enrolled up to 50 participants, six studies included 50–100 participants, 21 studies included 100–300 participants, and four studies included more than 300 participants. Among the included articles, 26 studies included both male and female participants, seven studies included only female participants, and the rest did not provide information about the gender distribution. Most of the interventions were conducted in primary healthcare centres (*n* = 15) and hospitals (*n* = 14). The mean age of participants, as reported in 31 papers, was 49.1 (±8.29) years old, while the rest of the studies did not provide information about the mean age of participants.

Among the studies included in this review, the majority utilised a quasi‐experimental pre–post design (*n* = 20, with 14 lacking a comparison group and the remainder incorporating a comparison group), followed by RCTs (*n* = 15, including two three‐arm trials and the remainder being two‐arm trials). The interventions conducted in these studies were mainly conducted by healthcare providers in collaboration with nurses (*n* = 14), with two studies solely conducted by nurses (Agbaria et al. 2020; Al‐Haj Mohd et al. 2016).

The predominant intervention type across the studies included was educational (*n* = 33), emphasising various aspects of diabetes management, such as promoting a healthy lifestyle, diabetes self‐care practices (such as foot care and blood glucose monitoring), dietary management, weight reduction, blood pressure control, smoking cessation, regular medical check‐ups, physical activity and discussion of symptoms and risks associated with diabetes complications (Al‐Hamdan et al. 2019; Al Mazroui et al. 2009; Rashed et al. 2016; Wani et al. 2020). Some studies also incorporated medication adherence and compliance strategies (Al‐Haj Mohd et al. 2016), while others tailored their interventions based on international standards and cultural/religious contexts (Agbaria et al. 2020; Al‐Bannay et al. 2015). Additionally, certain studies involved adjusting and administering insulin or oral antidiabetic therapy alongside medication reconciliation and education (Al Mazroui et al. 2009; Wishah, Al‐Khawaldeh, and Albsoul 2015), as well as the application and use of glucose monitoring systems or telemonitoring devices (Al Hayek, Robert, and Al Dawish 2021; Al‐Ofi et al. 2019). The comparison groups, on the contrary, typically received standard lifestyle advice or nonpersonalised counselling from healthcare providers.

Regarding the intensity and duration of interventions, the mean number of educational sessions was 6, ranging from 3 to 20 sessions, although some studies did not provide this information (*n* = 13). The sessions lasted between 30 min and 4 h and were conducted weekly or twice a month (AlHaqwi et al. 2023; Alibrahim et al. 2021). Among the included studies, the majority of interventions had a duration of 3 months (*n* = 8). However, it is noteworthy that certain studies did not provide explicit information regarding the duration of the intervention. Additionally, several studies conducted educational sessions emphasising personal contact (Al‐Hamdan et al. 2019, 2021), while others incorporated reminders, phone calls or messages to bolster intervention adherence (Abaza, Marschollek, and Schulze 2017; Al‐Haj Mohd et al. 2016; Al‐Hamdan et al. 2019; Haddad et al. 2014; Jarab et al. 2012). Postintervention follow‐up periods ranged from immediate assessments to up to 36 months. The most common follow‐up period was 6 months (*n* = 18), while some studies had one postintervention follow‐up, and others had multiple time‐points of follow‐up.

### 3.2 Methodological Quality

The overall assessment indicated an intermediate level of quality. Most studies tended to score low on the adjusted Downs and Black's checklist (Downs and Black 1998), which evaluates various aspects of study quality. The quality scores ranged from 11 to 24 points out of 28, with an average of 17.94 (±3.82) points. It is worth mentioning that only three studies reported blinding of those measuring the main outcomes (Abaza, Marschollek, and Schulze 2017; Alfawaz et al. 2018; Munsour et al. 2020).

### 3.3 Study Outcomes

A total of 65 outcomes were reported across 35 studies. These outcomes were categorised into three groups: objective measures were evaluated in 29 studies, subjective measures were assessed in 19 studies, and women‐related outcomes were analysed in seven studies.

#### 3.3.1 Objective Measures

##### 3.3.1.1 Laboratory Measures

###### 3.3.1.1.1 Glycaemic Control

The majority of studies (*n* = 26) examined the impact of various interventions on HbA1c levels (Table 2). The results presented a mixed picture: three RCTs reported no significant differences in HbA1c compared with the control group at the three‐month follow‐up (Abaza, Marschollek, and Schulze 2017; Al‐Hamdan et al. 2019; Munsour et al. 2020). However, these studies found a significant decrease in HbA1c at the 6‐month follow‐up (Al‐Hamdan et al. 2019; Al‐Shookri et al. 2012; Jarab et al. 2012; Wishah, Al‐Khawaldeh, and Albsoul 2015), and significant improvements were reported at the 12‐month follow‐up (Al‐Arifi and Al‐Omar 2018; Alfawaz et al. 2018; Al Mazroui et al. 2009; Mohamed et al. 2013). In a 3‐arm RCTs comparing different interventions with a control group, significant improvements in HbA1c levels were observed within all groups following the interventions. However, no significant differences were found between the groups (Al‐Hamdan et al. 2021). Among the single‐group pre–post designs, a significant decrease in HbA1c was reported in the majority of studies (*n* = 9), with significantly different values observed at the 3‐month follow‐up (AlHaqwi et al. 2023; Shehab, Elnour, and Abdulle 2012). Moreover, the majority of studies (11 out of 16) that examined the influence of different interventions on fasting plasma glucose reported a significant decrease.

**TABLE 2:** Summary of lab measures and physical exams in studies examining the effect of interventions on diabetes management in Arabs with diabetes.

Author	Follow up	HbA1c	LDL	HDL	Triglycerides	Cholesterol	FPG	MetS	A/C ratio	Weight	BMI	Waist‐hip ratio	Waist circumference (cm)	HIPS	Systolic pressure	Diastolic pressure
Abaza, Marschollek, and Schulze 2017	3 mo	NS^c^					NS^c^			NS^c^						
Agbaria et al. 2020	6 mo	NS	NS	NS	−					−	−				−	NS
Al Mazroui et al. 2009	4, 8, 12 mo	− 4, 8, 12 mo	− 4, 8, 12 mo	+ 4, 8, 12 mo	− 4, 8, 12 mo		− 4, 8, 12 mo				− 4, 8, 12 mo −^c^ 12 mo				− 4, 8, 12 mo	− 4, 8, 12 mo
		−^c^ 12 mo	−^c^ 12 mo	+^c^ 12 mo	−^c^ 12 mo		−^c^ 12 mo								−^c^ 12 mo	−^c^ 12 mo
Al‐Arifi and Al‐Omar 2018	3, 6, 12 mo	‐ 3, 6, 12 mo	‐6 mo, NS 3, 12 mo	NS	NS	NS 3 mo ‐6, 12 mo	NS 3 mo ‐6, 12 mo			NS					NS 3, 6 mo, −12 mo	NS 3, 6 mo, ‐12 mo
Al‐Bannay et al. 2015	6 mo						NS			NS			NS	—		
Alfawaz et al. 2018	6, 12 mo	Int(A)NS 6, 12 mo		Int.(A)NS 6, 12 mo	Int.(A) NS 6, 12 mo	Int.(A) NS 6 mo, ‐12 mo	Int(A)‐6, 12 mo Int(A)‐^c^	Int(A)‐12 mo, Int(A)‐^c^		INT(A)NS 6 mo, ‐12 mo	INT(A) NS 6 mo, ‐12 mo		INT(A) NS 6, 12 mo		INT(A) NS 6 mo, ‐12 mo	
		Int(B) ‐6, 12 mo		Int.(B)NS 6, 12 mo	Int.(B) NS 6, 12 mo	Int.(B)NS 6, 12 mo	Int(B)‐6, 12 mo Int(B)‐^c^	Int.(B) ‐12 mo, Int(B)‐^c^		INT(B)‐6, 12 mo	INT(B)‐6, 12 mo		INT(B) NS 6, 12 mo		INT(B) NS 6, 12 mo	
		CG NS 6, 12 mo		CG NS 6, 12 mo	CG NS 6, 12 mo	CG NS 6, 12 mo										
Al‐Haj Mohd et al. 2016	6 mo	‐^c^														
Al‐Hamdan et al. 2019	3, 6 mo	NS 3 mo, ‐6 mo, ‐^c^ 6 mo	NS 3 mo, ‐6 mo, NS 6 mo	NS 3 mo, ‐6 mo, +^c^ 6 mo	NS 3 mo, ‐6 mo, NS^c^ 6 mo	NS 3 mo, ‐6 mo, ‐^c^ 6 mo	NS 3 mo, ‐6 mo, NS^c^ 6 mo			NS 3, ‐6 mo, NS^c^	NS 3, ‐6 mo, NS^c^	NS 3, ‐6 mo, NS^c^	NS 3,6 mo, NS^c^	‐3 mo, NS 6 mo, NS^c^	NS 3, 6 mo, ‐^c^ 6 mo	NS 3, 6 mo, Ns^c^ 6 mo
Al‐Hamdan et al. 2021	6 mo	Int.(A) ‐, NS^c^ Int.(B) ‐, NS^c^ CG ‐ NS^d^	Int.(A) ‐, NS^c^ Int.(B) NS, NS^c^ ‐^d^	Int.(A) ‐, NS^c^ Int.(B) NS, NS^c^ NS^d^	Int.(A) ‐, NS^c^ Int.(B) NS, NS^c^ NS^d^	Int.(A) ‐, NS^c^ Int.(B) NS, NS^c^ NS^d^	Int.(A) ‐, NS^c^ Int.(B) NS, NS^c^ NS^d^			Int.(A) ‐, ‐^c^ Int.(B) ‐, ‐^c^ ‐^d^	Int.(A) ‐, ‐^c^ Int.(B) ‐, ‐^c^ ‐^d^				Int.(A) ‐, ‐^c^ Int.(B) ‐, ‐^c^ ‐b	Int.(A) ‐, ‐^c^ Int.(B) NS, ‐^c^ ‐b
AlHaqwi et al. 2023	3, 6 mo	− 3,6 mo	−3,6 mo	NS	− 3,6 mo	− 3,6 mo	− 3,6 mo				NS				NS 3 mo, ‐6 mo	NS 3, 6 mo
Al Hayek, Robert, and Al Dawish 2021	3 mo	NS														
Alibrahim et al. 2021	6, 12 mo	NS^c^ 6 mo, −^c^ 12 mo														
Al‐Shookri et al. 2012	3, 6 mo	−3, 6 mo	NS	NS	‐6 mo		‐3, 6 mo			‐3, 6 mo	‐3 mo, N S6 mo		‐3 mo, NS 6 mo			
		NS^c^	NS^c^	NS^c^	−^c^ 6 mo		−^c^ 3 mo			NS^c^	NS^c^		−^c^ 6 mo			
Ba‐Essa et al. 2015	4 mo	—	NS	+	NS	NS	−			NS					NS	NS
		NS^c^	NS^a^	+^c^	NS^c^	NS^c^	−^c^									
Baynouna et al. 2010	2, 3, 4 years	NS^a^ 3 yr −^a^ 4 yr									^a^−				^a^− 2, 3 yr	^a^− 2, 3, 4 yr
		^b^− 3 yr NS^b^ 4 yr									2, 34 yr				^a^NS 4 yr	
Istepanian et al. 2014	6 mo	NS, NS^c^	NS	NS	NS		NS									
Jarab et al. 2012	6 mo	NS		NS	NS		NS				NS, NS^c^				−, −^c^	−, −^c^
		−^c^		NS^c^	−^c^		−^c^									
Jenhani et al. 2005	6 mo	—														
Khattab et al. 2007	2 yr	—	—								—	—			—	NS
Mahmoud et al. 2018	5 mo	—														
Mohamed et al. 2013	12 mo	−^c^	NS^c^	+^c^		NS^c^			−^c^		−^c^				NS^c^	NS^c^
Munsour et al. 2020	3, 6 mo	NS^c^ 3, 6 mo														
Rashed et al. 2016	NI	—			—	—	—			—	—					
Reed et al. 2001	12 mo					NS	NS, ‐^c^								NS, (NI^c^)	NS, (NI^c^)
Sadiya, Abdi, and Abusnana 2016	3, 12 mo	‐ 3, 12 mo					‐ 3, 12 mo			‐ 3, 12 mo	‐ 3 mo	‐ 3 mo	‐ 3 mo			
Shehab, Elnour, and Abdulle 2012	3, 6 mo	‐6 mo	NS	NS	NS	NS	—								NS	NS
Wani et al. 2020	6, 12 mo			NS 6, 12 mo	NS 6, 12 mo	NS 6, 12 mo	‐ 6, 12 mo			NS 6 mo, ‐12 mo, ‐^c^	NS 6 mo, ‐12 mo (NI^c^)				NS 6, 12 mo	NS 6, 12 mo
Wishah, Al‐Khawaldeh, and Albsoul 2015	6 mo	—^c^	NS^c^	NS^c^	NS^c^	NS^c^	—^c^				NS					
Haddad et al. 2014		—														

*Note:* + Indicates an increase in the outcome variable. − Indicates a decrease in the outcome variable.

Abbreviations: A/C ratio, albumin creatinine ratio; BMI, body mass index; CG, control group; FPG, fasting plasma glucose; HbA1c, haemoglobin A1c; HDL, high‐density lipoprotein; Int(A), intervention A; Int(B), intervention B; LDL, low‐density lipoprotein; MetS, metabolic syndrome; mo, months; NI, no information; NS, not significant; yr, years.

^a^ Participants with diabetes.

^b^ Participants with diabetes and hypertension.

^c^ Compared with usual care.

^d^ Compared with another intervention.

###### 3.3.1.1.2 Lipid Profile

Approximately half of the studies included in this systematic review examined the impact of different interventions on the full or partial lipid profile (*n* = 17). Two RCTs reported a significant decrease in low‐density lipoprotein in the intervention group compared with the control group (Al‐Hamdan et al. 2021; Al Mazroui et al. 2009), while two single‐group pre–post studies also found a significant decrease in low‐density lipoprotein (AlHaqwi et al. 2023; Khattab et al. 2007). Four studies reported a significant influence on high‐density lipoprotein levels (Al‐Hamdan et al. 2019; Al Mazroui et al. 2009; Ba‐Essa et al. 2015; Mohamed et al. 2013). Regarding total cholesterol and triglyceride levels, half of the studies demonstrated a significant reduction, with most of these studies being pre–post designs. However, the majority of RCTs did not find a significant influence on total cholesterol in the intervention group compared with the control group (Al‐Hamdan et al. 2019; Mohamed et al. 2013; Wishah, Al‐Khawaldeh, and Albsoul 2015).

##### 3.3.1.2 Physical Examinations

###### 3.3.1.2.1 Weight and Body Mass Index

Out of the 13 studies included, 5 reported a significant reduction in weight, specifically after 6 and 12 months of intervention (Agbaria et al. 2020; Alfawaz et al. 2018; Al‐Hamdan et al. 2021; Sadiya, Abdi, and Abusnana 2016; Wani et al. 2020). In terms of body mass index (BMI), while most studies did not report a significant reduction in BMI at the 6‐month follow‐up period, a significant reduction was found at the 12‐month follow‐up period (Alfawaz et al. 2018; Al Mazroui et al. 2009; Mohamed et al. 2013; Wani et al. 2020), with sustained effects noted even after 4 years (Baynouna et al. 2010). Additionally, while some studies demonstrated a significant influence on waist circumference in the intervention group at 3 months compared with the control group, this effect was not observed at the 6‐month follow‐up period (Al‐Hamdan et al. 2019; Al‐Shookri et al. 2012).

###### 3.3.1.2.2 Blood Pressure

Most studies reported no significant change in either systolic or diastolic blood pressure (Agbaria et al. 2020; Ba‐Essa et al. 2015; Mohamed et al. 2013; Reed et al. 2001; Shehab, Elnour, and Abdulle 2012; Wani et al. 2020). However, one study was notable for demonstrating a significant decrease in both systolic and diastolic blood pressure at all follow‐up points. This particular study was a 2‐arm RCTs that implemented a 12‐month intervention involving pharmaceutical and lifestyle education (Al Mazroui et al. 2009). Furthermore, three studies indicated a significant decrease in systolic blood pressure but not in diastolic blood pressure (Alfawaz et al. 2018; Al‐Hamdan et al. 2019; AlHaqwi et al. 2023). On the contrary, two intervention studies reported no significant decrease in systolic blood pressure at 3‐ and 6‐month follow‐up, but a significant decrease was observed at the 12‐month follow‐up period (Al‐Arifi and Al‐Omar 2018; Alfawaz et al. 2018).

##### 3.3.1.3 Adherence to Diabetes Guidelines

Table 3 presents a comprehensive analysis of patient adherence to various diabetes management guidelines, encompassing a total of six studies. Among these studies, three consistently demonstrated a positive impact on conducting HbA1c blood tests at both 3‐ and 12‐month follow‐up periods (Abaza, Marschollek, and Schulze 2017; Reed et al. 2001, 2005). Additionally, two interventions showed a favourable influence on conducting cholesterol tests at the 12‐month follow‐up (Reed et al. 2001, 2005). Notably, one study with a longer follow‐up period of 36 months found significant improvements in conducting both HbA1c and cholesterol tests.

**TABLE 3:** Summary of adherence to diabetes guidelines in studies examining the effect of interventions on diabetes management in Arabs with diabetes.

Author	Follow‐up period	Conducting of HbA1c tests	Conducting fasting blood glucose tests	Conducting blood pressure	Conducting total cholesterol test	Ophthalmologist visit	Urine protein test	Adherence to follow‐up tests	Medication^a^ Adherence	Treatment^b^ Adherence
Reed et al. 2001	12 mo	+^c^, ^d^	+^c^, ^d^	+^c^, ^d^	+^c^, ^d^	+^c^, ^d^	+^c^, ^d^			
Reed et al. 2005	12 mo	+^c^, ^d^	NS^e^	NS^e^	+^c^	NS^e^	NS^e^			
Abaza, Marschollek, and Schulze 2017	3 mo	+^c^						+^c^	+^c^	+^c^
Munsour et al. 2020	3, 6 mo								NS^c^,3 mo, +^c^, 6 mo	
Al‐Haj Mohd et al. 2016	6 mo								+, +^c^	
Al‐Adsani et al. 2008	13, 24, 36 mo	+36 mo			+36 mo	+36 mo	+36 mo	+36 mo		

*Note:* + Indicates an increase in the outcome variable. − Indicates a decrease in the outcome variable.

Abbreviations: NS, Not significant; mo, months.

^a^ Treatment adherence was assessed by using the Diabetes Self‐Care Inventory (SCI).

^b^ Medication adherence was assessed by the Morisky Medication Adherence Scale (MMAS‐4).

^c^ Compared with usual care.

^d^ Number of patients.

^e^ Percent of patients.

In terms of medication adherence, three studies explored its effects at 3‐ and 6‐month follow‐up periods (Abaza, Marschollek, and Schulze 2017; Al‐Haj Mohd et al. 2016; Munsour et al. 2020), and the interventions consistently demonstrated a positive impact on medication adherence. However, one study revealed a nonsignificant impact of the intervention at the 3‐month follow‐up.

##### 3.3.1.4 Diabetes‐Related Cardiac Complications

Diabetes‐related cardiac complications were examined in only two studies. In the RCT conducted by Al Mazroui et al. (2009), a noteworthy reduction in the risk of coronary heart disease was observed at the 12‐month follow‐up. The reduction was assessed using the British National Formulary and Framingham method to estimate changes in the 10‐year coronary heart disease risk score. Conversely, in a quasi‐experimental single‐group pre–post study by Shehab, Elnour, and Abdulle (2012), no significant difference in microvascular complications was reported after 6 months. However, during the same 6‐month follow‐up, a significant reduction in macrovascular complications was observed.

#### 3.3.2 Subjective Measures

##### 3.3.2.1 Patient‐Reported Outcomes

A total of 12 studies were included in the evaluation of patient‐reported outcomes (Table 4). Among those that utilised different knowledge scales, six out of seven reported a significant increase in diabetes knowledge levels at 3‐ and 6‐months follow‐up periods (Abaza, Marschollek, and Schulze 2017; Al‐Bannay et al. 2015; Ba‐Essa et al. 2015; Mohamed et al. 2013; Rashed et al. 2016). However, one study, with a longer follow‐up period of 12 months, found no significant association between the intervention and diabetes knowledge (Reed et al. 2001). Regarding anxiety levels, as measured by the Hamilton scale and the Depression Anxiety Stress Scale (DASS‐42), significant changes were observed at the 6‐month follow‐up period (Jenhani et al. 2005). However, no significant reduction in anxiety levels was reported at 2 weeks and 3 months (Al‐Haj Mohd et al. 2016).

**TABLE 4:** Summary of patient‐reported outcomes in studies examining the effect of interventions on diabetes management in Arabs with diabetes.

Author	Follow‐up	Diabetes knowledge	Attitude score	Anxiety level	Self‐efficacy	Satisfaction	Psychological Well‐being	Bodily pain	General health	Mental health	Physical functioning	Role‐emotional	Role‐physical	Vitality	Social functioning
Rashed et al. 2016	NI	+													
Mohamed et al. 2013	12 mo	+, +^e^	+, +^e^												
Ba‐Essa et al. 2015	4 mo	+	+							+	+				
Al‐Bannay et al. 2015	6 mo	+				+^a^				NS^e^	NS^e^				
Jenhani et al. 2005	6 mo			−											
Reed et al. 2001	12 mo	NS				+^b^, ^e^, NS^c^, NS^d^									
Abaza, Marschollek, and Schulze 2017	3 mo	+^e^			+^e^	+, NS^b^									
Albikawi, Petro‐Nustas, and Abuadas 2016	2 wk, 3 mo			NS			NS 2 wk +^e^ 3 mo								
Mahmoud et al. 2018	5 mo						+^e^, +	NS	+		NS	+	NS	+	NS
Shehab, Elnour, and Abdulle 2012	3, 6 mo					+3, 6 mo^b^									
Al Mazroui et al. 2009	4, 8, 12 mo							+^e^	+^e^	+^e^	+^e^	+^e^	+^e^	+^e^	+^e^
Abduelkarem and Sackville 2008	3, 6, 24 mo							NS	−	NS	+	NS	+	+	NS

*Note:* + Indicates an increase in the outcome variable. − Indicates a decrease in the outcome variable.

Abbreviations: mo, months; NS, not significant; wk, weeks.

^a^ Life satisfaction.

^b^ Diabetes care satisfaction.

^c^ Diabetes treatment satisfaction.

^d^ Diabetes understanding satisfaction.

^e^ Compared with usual care.

Consistency in improvements in patient satisfaction was found in three studies indicating an improvement at various follow‐up periods. Specifically, Al‐Bannay et al. (2015) found higher life satisfaction, while other studies reported increased satisfaction regarding diabetes care (Reed et al. 2001; Shehab, Elnour, and Abdulle 2012). Concerning well‐being, no significant changes were observed at the short follow‐up period of 2 weeks (Al‐Haj Mohd et al. 2016), but significant reductions were reported at longer follow‐up periods of 3 and 5 months (Al‐Haj Mohd et al. 2016; Mahmoud et al. 2018). Finally, regarding the different domains of the quality‐of‐life scale, only the RCT reported improvements in all dimensions of the quality‐of‐life scale postintervention compared with control groups (Al Mazroui et al. 2009). Interestingly, the vitality dimension consistently improved across studies (Abduelkarem and Sackville 2008; Al Mazroui et al. 2009; Mahmoud et al. 2018).

##### 3.3.2.2 Lifestyle Modification

The evaluation of the intervention effect on physical activity included eight studies (Appendix S2). Among these studies, six reported a significant positive effect of the intervention, evident at both short and long‐term follow‐up periods (Abduelkarem and Sackville 2008; Agbaria et al. 2020; Ba‐Essa et al. 2015; Jarab et al. 2012; Mohamed et al. 2013; Wishah, Al‐Khawaldeh, and Albsoul 2015). Additionally, five studies assessed the general diet outcomes, with three of them reporting a significant effect of the intervention on dietary behaviours (Abduelkarem and Sackville 2008; Jarab et al. 2012; Wishah, Al‐Khawaldeh, and Albsoul 2015). These successful interventions for physical activity and general diet utilised various approaches, combining interventions with programme managers who maintained continuous contact with participants through WhatsApp, telephone calls, brochures and reminders following the intervention completion (Abduelkarem and Sackville 2008; Agbaria et al. 2020; Jarab et al. 2012; Wishah, Al‐Khawaldeh, and Albsoul 2015). Moreover, pedometers were used as a motivating tool to encourage participants to engage in more physical activity.

#### 3.3.3 Women‐Related Outcomes

##### 3.3.3.1 Outcomes for Women With Prediabetes or Diabetes

Five studies aimed to investigate the effectiveness of interventions among Arabic women with prediabetes or diabetes (Agbaria et al. 2020; Al‐Bannay et al. 2015; Al‐Hamdan et al. 2019, 2021; Sadiya, Abdi, and Abusnana 2016). These interventions primarily focused on providing advice on healthy nutrition and physical activity. Interestingly, these interventions had a significant impact on key outcomes, such as reducing HbA1c levels and BMI (Al‐Hamdan et al. 2019, 2021) and improving diabetic treatment behaviour, including carbohydrate intake and general diet modifications (Agbaria et al. 2020; Al‐Hamdan et al. 2019, 2021).

##### 3.3.3.2 Gestational Diabetes Mellitus Outcomes

Only two studies evaluated interventions aimed at improving outcomes among pregnant Arab women with gestational diabetes mellitus (GDM). In one study, women with GDM received counselling on nutrition and exercise and were subsequently followed up through their health centre (Utz et al. 2018). The other study provided participants with a telemonitoring device to monitor blood sugar and weight gain (Al‐Ofi et al. 2019). Both studies consistently demonstrated that the interventions led to significantly less weight gain compared with the control group. However, aside from this outcome, most other measures showed no significant differences between the intervention and control groups (Appendix S3).

#### 3.3.4 A Summary of Interventions Effectiveness

Due to the heterogeneity of the studies, which included variations in study design (some lacking a control group), interventions, duration and intensity, synthesising information proved challenging. Yet, based on the RCTs included in the current review, some conclusions about the effectiveness of the interventions can be made. For example, personal contact with patients through individualised lifestyle modifications, patient‐centred education, personalised advice, feedback, motivational conversations and telephone calls were associated with positive objective outcomes for the intervention group compared with the control group, such as improved HbA1c values and fasting blood glucose values (e.g., Al‐Hamdan et al. 2021; Al Mazroui et al. 2009; Jarab et al. 2012; Mohamed et al. 2013; Wishah, Al‐Khawaldeh, and Albsoul 2015). These intervention methods were also associated with greater physical activity in the intervention group compared with the control group (Jarab et al. 2012; Wishah, Al‐Khawaldeh, and Albsoul 2015). In contrast, studies based on nonpersonalised remote monitoring and social media showed no significant improvements for the intervention group compared with the control group (e.g., Abaza, Marschollek, and Schulze 2017; Al‐Hamdan et al. 2019; Istepanian et al. 2014). Another example is the use of weekly contact, either in person via phone or remotely via text, which was associated with higher adherence to medication in the intervention group compared with the control group (Abaza, Marschollek, and Schulze 2017; Al‐Haj Mohd et al. 2016). Additionally, interventions that included nutritional and physical activity advice had a positive impact on body mass index and systolic blood pressure among women (Al‐Hamdan et al. 2019, 2021).

## 4 Discussion

This systematic review is the first to systematically map all studies examining the effects of interventions on diabetes management among Arabs, particularly Arab women. It captures a diversity of outcomes, including objective, subjective and women‐related measures. Despite the methodological diversity and heterogeneity of the studies, some interventions can be highlighted as beneficial in improving diabetes management among Arabs.

Due to the fact that HbA1c is not just an objective measure of current health status but also a critical indicator of long‐term lifestyle changes and the potential reduction in diabetes‐related complications (Butalia et al. 2024; Lupu et al. 2022), it is not surprising that it was the primary outcome examined among the studies. Most studies found a significant reduction in HbA1c following the intervention at medium and long‐term follow‐up periods. The evidence of significant HbA1c reductions suggests that the interventions have been successful in promoting positive changes in participants' health behaviours, which is particularly encouraging for Arabs in the United Arab Emirates and Saudi Arabia, where HbA1c serves as a crucial predictor for lifestyle modification and the prevention of diabetes‐related complications (Al‐Hamdan et al. 2019; Al Mazroui et al. 2009). However, it is important to note that two RCTs reported no significant impact of the interventions on HbA1c levels. Several potential explanations for these null findings have been suggested, including relatively insufficient follow‐up times in the studies (Abaza, Marschollek, and Schulze 2017; Munsour et al. 2020), the provision of some form of intervention to participants in the control group, and continued contact between members of the intervention and control groups throughout the study (Abaza, Marschollek, and Schulze 2017). Additionally, factors such as low sample sizes, insufficient availability of healthcare diabetes specialists and clinicians in the region, and other contextual factors may have contributed to the lack of significant results in certain studies (Istepanian et al. 2014). The identified limitations provide valuable insights for future research in this area, highlighting the need for longer follow‐up periods, careful control of interventions in the control group and adequate sample sizes to enhance the validity and generalisability of the results. Moreover, efforts to strengthen the availability of healthcare specialists and clinicians in the region could contribute to the success of future interventions aiming to improve diabetes outcomes among Arabs.

In order to achieve beneficial results, it seems from the studies that certain intervention methods and content are preferred in the Arab population. Specifically, the effectiveness of personal contact with patients was evident, as it involved essential elements such as feedback, motivational conversations and patient‐centred education, in addition to the core intervention. Notably, individualised lifestyle self‐management and personalised advice played a significant role in improving HbA1c values, leading to positive outcomes (Alibrahim et al. 2021; Al Mazroui et al. 2009; Al‐Hamdan et al. 2021; AlHaqwi et al. 2023; Mohamed et al. 2013), aligning with the recommendations of the American Association of Diabetes Educators advocating for patient‐centred educational interventions based on patients' preferences, needs and expectations (Association of Diabetes Care and Education Specialists and Kolb 2021). Furthermore, interventions that incorporated reminders, feedback, motivational conversations and telephone calls demonstrated their impact by fostering improved treatment behaviours, including enhanced self‐monitoring of blood glucose and better adherence to prescribed therapies (Abaza, Marschollek, and Schulze 2017; Abduelkarem and Sackville 2008; Al‐Adsani et al. 2008; Jarab et al. 2012; Wishah, Al‐Khawaldeh, and Albsoul 2015).

Pharmaceutical administration, combined with medical education and patient adherence encouragement, alongside personalised approaches to healthy lifestyle self‐management, has demonstrated effectiveness in managing diabetes (Al‐Arifi and Al‐Omar 2018; Al Mazroui et al. 2009; Jarab et al. 2012; Wishah, Al‐Khawaldeh, and Albsoul 2015). These interventions empower patients by fostering autonomy in health decisions, which enhances disease management and medication adherence outcomes (Náfrádi, Nakamoto, and Schulz 2017; Sweileh et al. 2014). Medication adherence is recognised as a key determinant of therapeutic success in patients with diabetes mellitus. This underscores the importance of holistic approaches that integrate medical interventions with patient empowerment strategies to achieve comprehensive diabetes care.

According to the current systematic review, social media methods of intervention were less beneficial in the Arab population (Al‐Hamdan et al. 2021; Istepanian et al. 2014). This finding contrasts with the general population, where a systematic review highlighted the effectiveness of online social media interventions in improving treatment behaviour and clinical outcomes, either as stand‐alone interventions or in combination with face‐to‐face healthcare (Athiyah and Nita 2021). This disparity can be attributed to several factors, including cultural and social dynamics that favour face‐to‐face interactions and personalised advice, disparities in digital literacy and access, significant privacy concerns, stronger trust in traditional healthcare providers and the lack of culturally relevant content (Barqawi et al. 2023). Future studies should explore the use of social media interventions in different ways to assess their effectiveness further. However, given the current evidence, it may be advisable to focus on more culturally appropriate methods, as social media interventions may not be suitable for this population due to the aforementioned factors.

Regarding interventions focused on healthy nutrition and physical activity among women, positive impacts were observed, including weight loss and, in some cases, improvements in HbA1c levels and treatment behaviour (Agbaria et al. 2020; Al‐Bannay et al. 2015; Al‐Hamdan et al. 2019, 2021; Sadiya, Abdi, and Abusnana 2016). For example, a Saudi study explored tailored education models that enhanced glycaemic profiles in Arab women with prediabetes. It specifically targeted obstacles encountered by Saudi women, such as wearing abayas, gender segregation and indoor activities (Al‐Hamdan et al. 2019). These findings resonate with the recommendation to develop targeted programmes for women, as they are perceived as responsible for routine medical care and promoting healthy lifestyle choices within families (Bertran et al. 2021).

### 4.1 Limitations

The diversity among the studies in this review, in terms of research designs, sample sizes, intervention characteristics and outcomes, necessitated a narrative synthesis instead of a meta‐analysis. This diversity introduces several limitations that should be noted. First, diverse research designs beyond RCTs, including quasi‐experimental studies with or without a comparison group, could compromise the internal and external validity of the findings (Shadish, Cook, and Campbell 2002). However, the consistency of outcomes among different study designs indicates that findings from lower‐quality designs are supported by those from higher‐quality designs. Second, although studies have focused on a variety of outcomes, some outcomes were not tested. For example, no study has explored the impact of the intervention on wound healing, which is a critical issue among the diabetes population (Elkhalifa et al. 2024b). Third, outcomes explored in these studies encompassed the intertwined responsibilities of nurses and various practitioners, making it challenging to draw conclusions solely pertaining to nurses. Finally, only studies published in English were incorporated, although the primary author successfully screened studies in Arabic, enhancing the comprehensiveness of the literature pertaining to Arab patients with diabetes. Future research in this area should strive to address these limitations by adopting more rigorous study designs, larger sample sizes and standardised outcome measures to enhance the overall quality of evidence in interventions for Arab patients with diabetes.

## 5 Conclusion

This systematic review underscores that effective diabetes management among Arabs is significantly enhanced through healthy lifestyle self‐management, personalised care and tailored attention, complemented by pharmacological treatment and education. Reductions in HbA1c levels indicate successful promotion of positive health behaviours, particularly through interventions involving personal contact, feedback, motivational conversations and patient‐centred education. While social media interventions proved less effective, personalised nutrition and physical activity interventions that addressed cultural and social barriers, especially among women, showed positive impacts on weight loss, HbA1c levels and treatment behaviour. The findings provide valuable insights into effective intervention components that can contribute to improving diabetic outcomes in this population. By incorporating these components in future interventions, meaningful improvements in diabetes management can be achieved.

## Conflicts of Interest

The authors declare no conflicts of interest.

### Peer Review

The peer review history for this article is available at https://www.webofscience.com/api/gateway/wos/peer‐review/10.1111/jan.16423.

## Supporting information


Data S1.

Appendix S1.

Appendix S2.

Appendix S3.

## Data Availability

The data supporting the findings of this systematic review are available within the article and its supplementary materials. All data used in this review were extracted from previously published studies, which are cited and referenced in the manuscript.
