# Supplementary material for: The Effects of Diverse Interventions on Diabetes Management Among Arabs With Diabetes: A Systematic Review
Source: J Adv Nurs. 2024 Sep 5;81(3):1222–40. doi: 10.1111/jan.16423 (PMC11810500; doi:10.1111/jan.16423)
Supplement: Supplementary file 2 — Appendix S1. [file JAN-81-1222-s002.docx]

**Appendix 1.** Search Strategy in Three Databases

**PubMed Search Strategy**

("Diabetes Mellitus"[MeSH Terms]) AND (("Arabs"[MeSH Terms]) OR ("Algeria"[MeSH Terms] OR "Bahrain"[MeSH Terms] OR "Comoros"[MeSH Terms] OR "Djibouti"[MeSH Terms] OR "Egypt"[MeSH Terms] OR "Iraq"[MeSH Terms] OR "Jordan"[MeSH Terms] OR "Kuwait"[MeSH Terms] OR "Lebanon"[MeSH Terms] OR "Libya"[MeSH Terms] OR "Mauritania"[MeSH Terms] OR "Morocco"[MeSH Terms] OR "Qatar"[MeSH Terms] OR "Saudi Arabia"[MeSH Terms] OR "Somalia"[MeSH Terms] OR "Sudan"[MeSH Terms] OR "Syria"[MeSH Terms] OR "Tunisia"[MeSH Terms] OR "United Arab Emirates"[MeSH Terms] OR "Yemen"[MeSH Terms])) AND ("Program Evaluation"[MeSH Terms] OR "Health Promotion"[MeSH Terms] OR "Self Care"[MeSH Terms] OR "Health Behavior"[MeSH Terms] OR "Patient Education as Topic"[MeSH Terms] OR "Evaluation Studies as Topic"[MeSH Terms])

**CINAHL Search Strategy**

(MH "Diabetes Mellitus") AND ((MH "Arabs") OR (MH "Algeria") OR (MH "Bahrain") OR (MH "Comoros") OR (MH "Djibouti") OR (MH "Egypt") OR (MH "Iraq") OR (MH "Jordan") OR (MH "Kuwait") OR (MH "Lebanon") OR (MH "Libya") OR (MH "Mauritania") OR (MH "Morocco") OR (MH "Qatar") OR (MH "Saudi Arabia") OR (MH "Somalia") OR (MH "Sudan") OR (MH "Syria") OR (MH "Tunisia") OR (MH "United Arab Emirates") OR (MH "Yemen")) AND ((MH "Program Evaluation") OR (MH "Health Promotion") OR (MH "Self Care") OR (MH "Health Behavior") OR (MH "Patient Education"))

**Cochrane Library Search Strategy**

[mh "Diabetes Mellitus"] AND ([mh Arabs] OR [mh Algeria] OR [mh Bahrain] OR [mh Comoros] OR [mh Djibouti] OR [mh Egypt] OR [mh Iraq] OR [mh Jordan] OR [mh Kuwait] OR [mh Lebanon] OR [mh Libya] OR [mh Mauritania] OR [mh Morocco] OR [mh Qatar] OR [mh "Saudi Arabia"] OR [mh Somalia] OR [mh Sudan] OR [mh Syria] OR [mh Tunisia] OR [mh "United Arab Emirates"] OR [mh Yemen]) AND ([mh "Program Evaluation"] OR [mh "Health Promotion"] OR [mh "Self Care"] OR [mh "Health Behavior"] OR [mh "Patient Education"] OR [mh "Evaluation Studies as Topic"])
