# Supplementary material for: The Effects of Diverse Interventions on Diabetes Management Among Arabs With Diabetes: A Systematic Review
Source: J Adv Nurs. 2024 Sep 5;81(3):1222–40. doi: 10.1111/jan.16423 (PMC11810500; doi:10.1111/jan.16423)
Supplement: Supplementary file 3 — Appendix S2. [file JAN-81-1222-s003.docx]

Appendix **2.** Summary of Lifestyle Modification Subjective Outcomes in Studies Examining the Effect of Interventions on Diabetes Management in Arabs with Diabetes

| Author | Follow up | Energy Intake | Carboy drate(g) intake | Protein (g)intake | Fat (g)intake | Vegetables & Fruit (d/Avg) | Whole grains | Diet general | Physical activity | self-monitoring blood glucose | Foot care | Smoking | Fundus examination | health care use |
| --- | --- | --- | --- | --- | --- | --- | --- | --- | --- | --- | --- | --- | --- | --- |
| Al-Hamdan et al., 2019 | 3,6 months | -3,6mo  -^a*^ | -3,6mo,  -^a*^ | -3mo,  NS6mo,  -^a*^ | -3,6mo  NS^a^ |  |  |  | NS,  NS^a^ |  |  |  |  |  |
| Mohamed et al., 2013 | 12 months |  |  |  |  |  |  |  | +^a^ |  |  |  |  |  |
| Agbaria et al., 2020 | 6 months |  | **-** |  |  | NS | **-** | NS | + |  |  |  |  |  |
| Ba-Essa et al..,2015 | 4 months |  |  |  |  |  |  |  | +, +^a^ |  |  |  |  |  |
| Al-Bannay et Al., 2015 | 6 months |  |  |  |  |  |  |  |  | **-** |  |  |  |  |
| Khattab et al.,2007 | 2 years |  |  |  |  |  |  |  |  |  |  | **-** | + |  |
| Jarab et al.,2012 | 6 months |  |  |  |  |  |  | +^a^ | +^a^ | +^a^ | NS^a^ | NS^a^ |  |  |
| Al-Adsani et al., 2008 | 13,24,36 months |  |  |  |  |  |  |  |  |  | + | + | + |  |
| Al Hayek et al., 2021 | 3 months |  | **-** |  |  |  |  | NS | NS | NS |  |  |  | NS |
| Al-Hamdan et al.,2021 | 6 months | Int. (A)  -, -^a^  Int. (B)  -, -^a^ | Int. (A)  -, -^a^  Int. (B)  -, -^a^ | Int. (A)  -, -^a^  Int. (B)  -, -^a^ | Int. (A)  -, -^a^  Int. (B)  -, -^a^ |  |  |  |  |  |  |  |  |  |
| Abduelkarem & Sackville , 2009 | 3,6,24 months |  |  |  |  |  |  | -3,6mo,  NS24mo | +3,6 mo,  NS24mo | +3,24mo,  NS6mo | +3,6mo  NS24mo |  |  |  |
| Wishah et al., 2015 | 6 months |  |  |  |  |  |  | +^a^ | +^a^ | +^a^ | +^a^ |  |  |  |

Note: NS, Not significant; mo, months; Int(A), Intervention A; Int(B), Intervention B

+ Indicates an increase in the outcome variable

− Indicates a decrease in the outcome variable

^a^ Compared to usual care
