# Supplementary material for: The Effects of Diverse Interventions on Diabetes Management Among Arabs With Diabetes: A Systematic Review
Source: J Adv Nurs. 2024 Sep 5;81(3):1222–40. doi: 10.1111/jan.16423 (PMC11810500; doi:10.1111/jan.16423)
Supplement: Supplementary file 4 — Appendix S3. [file JAN-81-1222-s001.docx]

**Appendix 3.** Summary of Outcomes in Studies Examining the Effect of Interventions on Diabetes Management in Arab Pregnant Women with Gestational Diabetes Mellitus

| Author | Follow up | Mean birthweight | Compliance with referral to a specialist | Follow-up visits at health and referral centers | Maternal weight gain/week | Gestational age at delivery | Mode of delivery | Delivery complications | Macrosomia | Post Prandial Glucose | HbA1c | Oral glucose tolerance tests | Fasting Plasma Glucose |
| --- | --- | --- | --- | --- | --- | --- | --- | --- | --- | --- | --- | --- | --- |
| Utz et al., 2018 | Second trimester, up to 8 weeks post-partum | -, NS^a^ (1-26 days after delivery) | NS^a^ | +^a^ | -^a^ (weekly) | NS^a^ | NS^a^ | NS^a^ | -^a^ |  |  | NS^a*^(post-partum) | NS^a*^(post-partum) |
| Al-Ofi et al., 2019 | 24–28 weeks of gestation, end of pregnancy |  |  |  | -^a^ (weekly) |  |  |  |  | -, NS^a^(end of pregnancy) | NS^a^ (end of pregnancy) |  | NS, NS^a^ (end of pregnancy) |

Note: Hemoglobin A1C, HbA1c; Not significant, NS

+ Indicates an increase in the outcome variable

− Indicates a decrease in the outcome variable

^a^ Compared to usual care
